# Investigating the Trimethylaluminium/Water ALD Process on Mesoporous Silica by In Situ Gravimetric Monitoring

**DOI:** 10.3390/nano8060365

**Published:** 2018-05-24

**Authors:** Verena E. Strempel, Kristian Knemeyer, Raoul Naumann d’Alnoncourt, Matthias Driess, Frank Rosowski

**Affiliations:** 1 BasCat—UniCat BASF JointLab, Technische Universität Berlin, Hardenberstraße 36, 10623 Berlin, Germany; v.strempel@bascat.tu-berlin.de (V.E.S.); k.knemeyer@bascat.tu-berlin.de (K.K.); matthias.driess@tu-berlin.de (M.D.); frank.rosowski@basf.com (F.R.); 2 Institut für Chemie, Technische Universität Berlin, Straße des 17, Juni 135, 10623 Berlin, Germany; 3 Process Research and Chemical Engineering, BASF SE, Carl-Bosch-Straße 38, 67056 Ludwigshafen, Germany

**Keywords:** atomic layer deposition, ALD, in situ gravimetric, mesoporous silica, ligand exchange, fixed bed, particle coating

## Abstract

A low amount of AlO_x_ was successfully deposited on an unordered, mesoporous SiO_2_ powder using 1–3 ALD (Atomic Layer Deposition) cycles of trimethylaluminium and water. The process was realized in a self-built ALD setup featuring a microbalanceand a fixed particle bed. The reactor temperature was varied between 75, 120, and 200 °C. The self-limiting nature of the deposition was verified by in situ gravimetric monitoring for all temperatures. The coated material was further analyzed by nitrogen sorption, inductively coupled plasma-optical emission spectroscopy, powder X-ray diffraction, high-resolution transmission electron microscopy, attenuated total reflection Fourier transformed infrared spectroscopy, and elemental analysis. The obtained mass gains correspond to average growth between 0.81–1.10 Å/cycle depending on substrate temperature. In addition, the different mass gains during the half-cycles in combination with the analyzed aluminum content after one, two, and three cycles indicate a change in the preferred surface reaction of the trimethylaluminium molecule from a predominately two-ligand exchange with hydroxyl groups to more single-ligand exchange with increasing cycle number. Nitrogen sorption isotherms demonstrate (1) homogeneously coated mesopores, (2) a decrease in surface area, and (3) a reduction of the pore size. The experiment is successfully repeated in a scale-up using a ten times higher substrate batch size.

## 1. Introduction

Today, technological applications of materials with high surface aspect ratios are plentiful. Among these materials, nanoporous solids play an outstanding role for in many applications, e.g., catalysis, fuel cells, and membranes [1]. However, porous materials can feature undesirable surface properties and a precise surface modification or functionalization is required for further application [2]. Especially in heterogeneous catalysis, surface deposits in a submonolayer regime can act as active sites or promoters [3,4]. In consequence, surface coating techniques for porous structures with high uniformity and control over the elemental composition, thickness, and morphology need to be developed. The most promising candidate for the ultra-thin coating of complex high aspect ratio structures is Atomic Layer Deposition (ALD) [5,6]. 

Traditionally ALD is a thin-film deposition technique that evolved from Chemical Vapor Deposition (CVD) [7]. Just as in CVD, ALD uses two, highly reactive, vapor-phase precursors to form a deposit on a surface. In contrast to CVD, those precursors are separated by time or space. The substrate is sequentially exposed to an overdose of metal and/or non-metal precursors by applying sufficient inert gas purging in between following an A-purge-B-purge-A-purge-B-etc. sequence. The precursor molecules react with surface sites (e.g., OH groups) until saturation. This leads to the unique self-limiting nature of ALD, as the reaction terminates once all the reactive sites on the surface are consumed and/or sterically shielded [8]. By repeating the complete cycle, the thickness of the grown film can be controlled to fractions of a nanometer. The achieved film thickness is therefore solely defined by the reaction temperature, pressure, and the number of reaction cycles [9].

By using ALD for the coating of porous powders in a submonolayer regime, two main challenges have to be handled: (1) The coating of powders is demanding, as ALD is mainly established for flat substrates in absence of pore diffusion phenomena [10,11], e.g., wafers; and (2) the first ALD cycles differ in mechanism and growth from the following cycles since they occur mostly on the surface of the original substrate [12]. 

Here, we report about the growth of a very thin AlO_x_ layer on an unordered, mesoporous SiO_2_ powder. The film is built-up by the sequence of trimethylaluminium (TMA) and water on the surface of the heated substrate powder. We have chosen AlO_x_ (Al_2_O_3_) as the coating material, because the AlO_x_ process is one of the most-understood ALD processes in literature [13]. The deposition of this material on various substrates at different temperatures is studied with many techniques. The process features a straightforward chemistry between the highly reactive precursor and mainly the OH groups on the surface. In addition, the obtained films possess excellent thermal and chemical stability. The goal of this paper is to provide a comprehensive study of the modification of the SiO_2_ surface with a (sub)monolayer of AlO_x_. To achieve such a low loading, the highest number of ALD cycles is three. The mesoporous SiO_2_ starting material (Davisil, grade 636, 506 m^2^ g^−1^) is a widely used support material for heterogeneous catalysts and therefore of particular interest for surface modifications [14,15].

The studies are conducted in a self-built versatile ALD setup using fixed particle beds, presented elsewhere [15]. The fixed-bed offers an excellent gas-solid contact. This reactor geometry is highly suitable for the deposition of very thin films on high surface area materials [16]. Our setup is equipped with a magnetic suspension balance, which measures in situ the mass changes during the ALD. Usually, ALD on flat substrates can be in situ monitored by using a Quartz Crystal Microbalance (QCM). A QCM confirms, in general, the self-limitation of an ALD process, but gives no information about the saturation of the specific sample itself, which is usually placed before the QCM. The in situ gravimetric monitoring is an effective and non-destructive way of studying the self-limitation of the ALD process and measures the deposited mass directly on the powder substrate of interest. The gravimetric data, combined with further chemical and physical analysis of the samples, helps to gain new insights of the first couple of ALD cycles on the porous powder. The determined ALD parameters were transferred and the coating was repeated with a 10× higher substrate batch size to investigate the scale-up possibilities of fixed beds for ALD. In addition, we discuss the experimental data with regard to a possible AlO_x_ monolayer and its structure and dimension.

## 2. Experimental Section

We used an amorphous, unordered, and mesoporous SiO_2_ powder (Davisil, Grade 636) as our starting material and coated it with a very thin AlO_x_ deposit via ALD. The SiO_2_ is a well-known support material especially for heterogeneous catalysts, as it is commercially available in large amounts with varied surface areas, mesh sizes, and average pore diameters.

Trimethylaluminium (TMA) served as the precursor and H_2_O as the reactant (second precursor). The correct ALD parameters and the self-limiting nature of the reactions were determined in the thermal balance. That followed a scale-up in a large fixed bed reactor. The samples were characterized with the given methods below.

### 2.1. Chemicals

Silica powder (SiO_2_, high-purity grade ≥ 99%; Davisil Grade 636; average pore size 60 Å, 35–60 mesh particle size, Sigma-Aldrich, (St. Louis, Mo, USA), 506 m^2^ g^−1^) was used as the substrate. Trimethylaluminium (Al(CH_3_)_3_, TMA, elec. gr., 99.999% Al) and water (H_2_O, CHROMASOLV^®^, for HPLC, Riedel-de Haën/Honeywell Specialty Chemicals Seelze GmbH, Seelze, Germany) served as precursors and were used without further purification. High purity N_2_, Ar, and He (99.999%) acted as carrier and purging gases.

### 2.2. AlO_x_ ALD Experimental

AlO_x_ coating experiments were conducted under reactor temperatures between 75–200 °C with 1–3 cycles in a self-built ALD setup comprising a magnetic suspension balance and a large fixed-bed reactor. A detailed setup description and experimental procedure is given elsewhere [15]. The amount of initial substrate varied between 100–600 mg. In contrast to many other ALD setups, the precursors were not pulsed, but sequentially introduced into the reactor in constantly flowing inert carrier gas. As precursors served TMA and H_2_O in the sequence TMA (in N_2_)-Ar purge-H_2_O (in He)-Ar purge. The setup was operated at atmospheric pressure under constant flow conditions with a total gas flow of 50 mL min^−1^. The setup featured an inert gas line for purging (Ar) and two gas lines flowing through different precursor saturators (N_2_ for TMA and He for H_2_O). By a system of switching valves, the different gases were distributed to the ALD reactor or to bypass lines. This design enabled a convenient switching between different gas atmospheres with or without precursors in the ALD reactor while maintaining a constant overall flow and a constant precursor delivery. The TMA saturator was operated with 40 mL min^−1^ N_2_, which was diluted in additional 10 mL min^−1^ N_2_ for the reactor temperatures of 75 and 200 °C. At 120 °C the flow was reduced to 25 mL min^−1^ N_2_ in the saturator and 25 mL min^−1^ N_2_ dilution. Typical half-cycle times lay in the range of hours depending on substrate mass. Mass changes were in situ monitored by gravimetry every 25 min for 5 min during the experiment. Atmosphere changes in the reactor, precursor, and byproducts were monitored with an online mass spectrometer. The precursor sequence was finished when the balance reached equilibrium and the MS showed a steady state. Valve switching between the ALD sequence was done manually.

In addition, a scale-up experiment was conducted at 200 °C. The ALD parameters and settings were transferred from the experiments in the balance. The fixed bed was divided by quartz wool into three segments.

### 2.3. Characterization Methods

N_2_ physisorption measurements were performed at liquid N_2_ temperature on a Quantachrome Autosorb-6B analyzer (Quantachrome GmbH & Co. KG, Odelzhausen, Germany). Prior to the measurement, the samples were degassed in dynamic vacuum at 150 °C for 2 h. Full adsorption and desorption isotherms were measured. The specific surface area S_BET_, the pore size, and pore volume were calculated according to the multipoint Brunauer-Emmett-Teller method (BET) and Barrett-Joyner-Halenda (BJH) method, respectively. 

Inductively coupled plasma-optical emission spectroscopy (ICP-OES, Varian 720-ES, Varian Inc., Palo Alto, CA, USA) was used to determine the Al content of the samples. Solutions from the powders were prepared via microwave-assisted acid digestion using concentrated H_2_SO_4_ (95%) and concentrated H_3_PO_4_ (85%). The sample was treated at 300 °C for 30 min after 12 h of stirring. The spectroscope was five-point calibrated with a commercially available, diluted standard for Al. 

Powder X-ray diffraction (XRD) measurements were performed in Bragg-Brentano geometry on a Bruker AXS D8 Advance II theta/theta diffractometer (Bruker AXS GmbH, Kalrsuhe, Germany), using Ni filtered Cu Kα radiation and a position sensitive energy dispersive LynxEye silicon strip detector. The sample powder was filled into the recess of a cup-shaped sample holder, the surface of the powder bed being flush with the sample holder edge.

High-resolution transmission electron microscopy (HRTEM) images were recorded at the C_s_-corrected TITAN 80–300 Berlin Holography Special TEM (Thermo Fisher Scientific Inc., Waltham, MA, USA), operated at 300 kV.

Attenuated total reflection Fourier transformed infrared spectroscopy (ATR-FTIR) was performed on a Bruker ALPHA FT-IR spectrometer (Bruker Optik GmbH, Ettlingen, Germany) using a permanently adjusted ROCKSOLID interferometer and a DTGS detector (deuterated triglycinsulphate). The setup was equipped with a single reflection diamond crystal ATR accessory (PLATINUM-ATR). Samples were crushed in a mortar and uniformly distributed on the 2 mm × 2 mm crystal. Each spectrum was acquired by integration of 24 interferograms with a resolution of 4 cm^−1^ and correcting with atmospheric background.

Elemental analyses were performed on a Thermo Finnigan ThermoFlashEA 1112 Elemental Analyzer with a MAS 200 autosampler (Thermo Fisher Scientific Inc., Waltham, MA, USA) for solid samples. The gaseous compounds are separated via gas chromatography (GC) and detected by a thermal conductivity detector (TCD). Values were averaged over two separate measurements.

## 3. Results and Discussion

We successfully deposited a very thin AlO_x_ layer with different numbers of TMA and H_2_O cycles at three substrate temperatures on a mesoporous SiO_2_ powder. Usually, ALD is used to produce nm thick layers with 50 and more cycles. The highest cycle amount conducted in this study was three, as we were interested in ALD in a submonolayer regime. Samples 200.1c, 200.2c, 200.3c, 120.3c, and 75.3c were synthesized in the thermal balance reactor and the mass gain was measured in situ. The sample family consisted of six samples including the reference SiO_2_. To give a good general overview, the results for those samples are presented in Table 1. The following sections will be discussed and results presented in detail for thematically grouped samples. The samples produced in the scale-up fixed bed reactor FB.top, FB.middle, and FB.bottomare are presented in Table 2 and are discussed separately.

The reference SiO_2_ is an often-used support material and was characterized previously with XRD, N_2_ adsorption for BET/BJH calculations, and ICP-OES. In addition, thermogravimetric analysis combined with mass spectrometry (TG-MS) are presented elsewhere [15]. The results verify that all physisorbed water on the SiO_2_ is removed by sufficient drying at 72 °C and no further condensation of surface groups occurred.

Usually, the intrinsic self-saturation of the ALD half-reactions is proven by analyzing the amount of surface coating as a function of dosing time. The deposited amount will be independent from the dosing time, when the surface is saturated. This requires at least five experiments or more and the corresponding analytics [17]. Here, we used a setup with in situ gravimetric monitoring and needed only one experiment. The self-limitation of the process revealed itself during the experiment. Our samples, prepared in the balance, showed complete saturation curves, hence self-termination, for all temperatures and all cycles. To stay in the scope of this manuscript, the full curves are shown in the Supplementary Materials and solely the relative mass gains are discussed in the following sections. In addition, the interested reader can find examples for the QMS monitoring elsewhere [15].

### 3.1. Influence of Cycle Number

**Figure 1:**
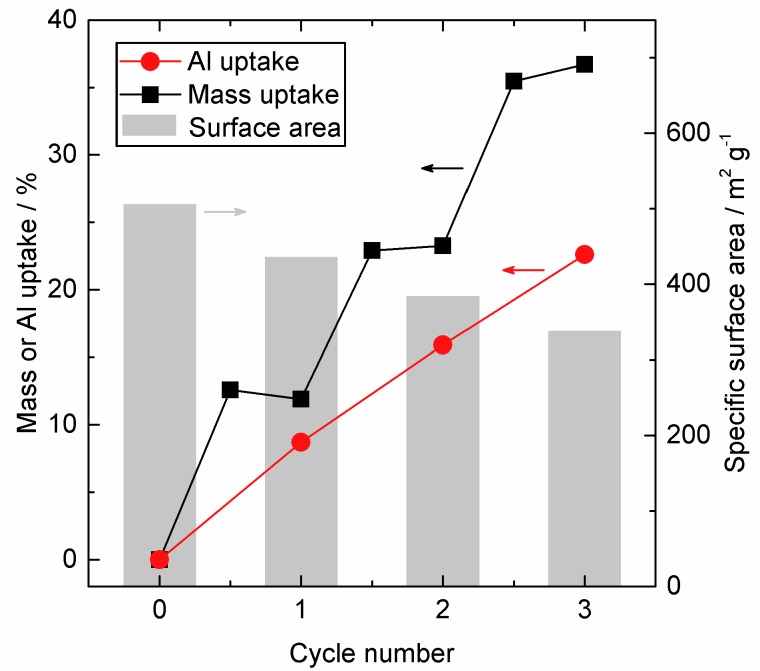
Specific surface area (grey, background, right axis), mass uptake in wt % (black, left axis) and uptake Al wt % (red, left axis) for the SiO_2_ reference and after 1–3 ALD AlO_x_ cycles at 200 °C.

At the reactor temperature of 200 °C samples after one, two, and three cycles were prepared and further analyzed to study the influence of the cycle number on the coating and the pore structure of the SiO_2_. The relative mass gain per cycle is presented together with the calculated BET surface area and the Al uptake in wt % determined with ICP-OES in Figure 1.

Beside the saturation curves (for details see Supplementary Materials), the in situ gravimetry gives two pieces of information: (1) the total mass gain, which was around 12–13% for each cycle, and (2) the uptake, which is distributed differently between the two half cycles from cycle to cycle. In the first cycle, the H_2_O half-cycle led to a slight mass loss of −0.7%. With increasing cycle number, the contribution of the H_2_O half-cycle to the total mass gain increased from 0.4% for cycle two and 1.2% for cycle three. 

For an interpretation of this data, it is necessary to have a look at the surface reactions during the ALD. It is known, that TMA can undergo different chemisorption mechanisms on the surface, shown in Equations (1) and (2) (with 1 ≤ *x* ≤ 3) [17].

(1) $$x\;\|O-H+\mathrm{Al}{\left({\mathrm{CH}}_{3}\right)}_{3}\;\left(g\right)\to \;{\left(\|O-\right)}_{x}\mathrm{Al}{\left({\mathrm{CH}}_{3}\right)}_{3-x}\;+x{\;\mathrm{CH}}_{4\;}\left(g\right)$$

(2) $${\left(\|O-H\right)}_{x}\mathrm{Al}{\left({\mathrm{CH}}_{3}\right)}_{3-x}\;+\left(3-x\right){\;H}_{2}O\to \;{\left(\|O-\right)}_{x}\mathrm{Al}{\left(\mathrm{OH}\right)}_{3-x}\;+\left(3-x\right){\;\mathrm{CH}}_{4\;}\left(g\right)$$

The molecule has three methyl groups and, if we leave dissociation and association mechanisms out of consideration for convenience, it can react via a single exchange (*x* = 1), a double ligand exchange (*x* = 2), or a triple ligand exchange (*x* = 3). The following H_2_O cycle hydrolyzes the remaining methyl groups, releasing them as methane and repopulates the surface with hydroxyl groups (Equation (2)). Based on these equations, the additional molar mass per precursor molecule and the contribution from the H_2_O cycle can be calculated: 60 g mol_Pre_^−1^ for single exchange with 6% mass gain from H_2_O, 42 g mol_Pre_^−1^ for double exchange with 5% from H_2_O, and 24 g mol_Pre_^−1^ for triple ligand exchange without contribution from H_2_O [18] (see as well Figure S4 in Supplementary Materials).

Coming back to our data, the experimental molar mass for the adsorbed Al species can be calculated with the mass gain and Al content in the sample. The results show a clear trend to increasing mass with increasing cycle number: 39 g mol_Pre_^−1^ for cycle one, 43 g mol_Pre_^−1^ for cycle two, and 51 g mol_Pre_^−1^ for the last cycle. This brief calculation correlates with predominantly single and double ligand exchange reactions in our ALD reaction with a larger contribution of single ligand exchanges with increasing cycle number. Yet, the observed mass contributions from H_2_O were too low to directly fit double and single ligand exchanges. This can be rationalized with possible condensation reactions of two hydroxyl group, either both emerging on Al atoms or one Al-OH group and one Si- OH group. This condensation leads to an additional mass loss and can cancel out other mass gains. In general, the change of the surface reactions with cycle number is thoroughly anticipated, as the surface composition changes during the first ALD cycles from a SiO_2_ to an AlO_x_ surface [19]. A repetitive cycle with equal mass gains/Al gains will begin, as soon as a reproducible surface is formed. 

Furthermore, Figure 1 illustrates the linearly increasing amount of Al with increasing cycle number. For the first cycle, almost all mass gain was built up by Al itself and consequently only a small amount was contributed by additional O and OH incorporation. Though this contribution grew with increasing cycle number. This confirms the previous conclusion of potential condensations of OH groups and a higher measurable impact of the H_2_O treatment in the following cycles. For comparison, the mass ratio in pure Al_2_O_3_ is 53:47 (Al:O), i.e., would lead to 53 wt % mass gain by Al (without considering potential H atoms).

**Figure 2:**
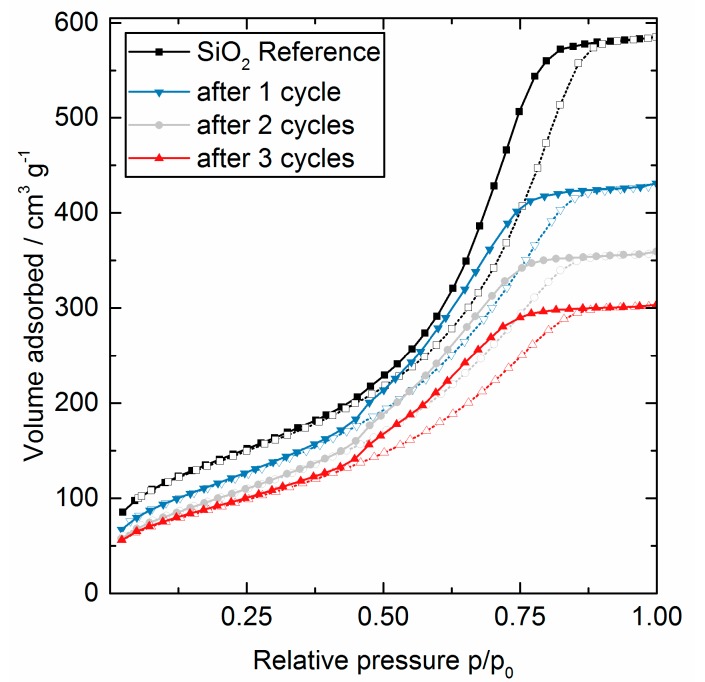
N_2_ adsorption (hollow symbols, dotted line) and desorption (full symbols, solid line) isotherms for SiO_2_ coated with AlO_x_ via ALD using TMA and H_2_O at 200 °C. Shown are the reference SiO_2_ (black, ■), after one cycle (blue, ▼), after two cycles (grey, ●), and after three cycles (red, ▲).

**Figure 3:**
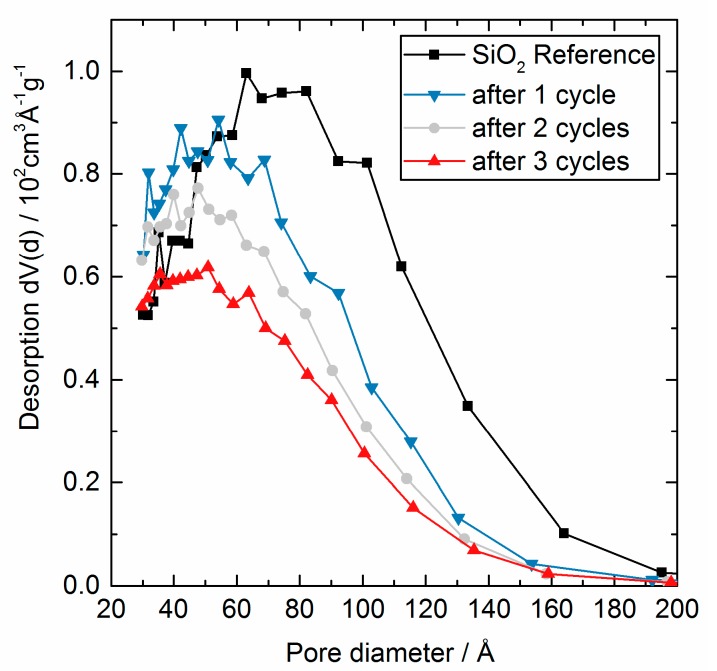
Differential pore volume distribution determined by N_2_ desorption, calculated applying BJH theory for SiO_2_ coated with AlO_x_ via ALD using TMA and H_2_O at 200 °C. Shown are the graphs for reference SiO_2_ (black, ■), after 1 cycle (blue, ▼), after 2 cycles (grey, ●), and after 3 cycles (red, ▲).

Nitrogen physisorption measurements were performed to analyze the effect of the AlO_x_ deposit on the porous structure of the support. The full desorption and adsorption isotherms are presented in Figure 2 and the calculated differential pore size distribution in Figure 3. The distribution was calculated using the method proposed by Barrett, Joyner, and Halenda (BJH) [20]. 

All samples gave an H_1_ type hysteresis loop, with parallel adsorption and desorption branches, due to the capillary condensation of N_2_ in mesopores. In addition, the data shows a clear decrease in the step height of the isotherms as a function of cycle number, a general downward shift, and an accompanied slight horizontal shift to lower relative pressures, better seen in the resulting decreasing pore size distribution. The combination of the two shifts and reduction in step height verifies a conformal coating of the pores. All other possible growth mechanisms in the pores can be excluded. Those mechanisms would lead to other shaped isotherms, e.g., a gradual filling of the pores with AlO_x_ and/or the covering of the pore entrance do not result in a horizontal shift/a lower pore size [21]. The formation of large AlO_x_ particles in the pores would lead to an H_2_ type isotherm, characteristic of ‘ink bottle’ type of pores [22].

The BET surface area as a function of cycle number is shown in Figure 1 and the absolute values are as well presented in Table 1. The surface area decreased from 506 m^2^ g^−1^ to 337 m^2^ g^−1^ during three cycles due to the decrease of the pore volume, when the pore size was continuously reduced. In addition, the covering of micropores can contribute to the observed surface area reduction, as the decreasing intercept of the adsorbed volume may indicate. Literature suggests that an increasing cycle number would influence the surface area less sharply, once all micropores and mesopores are blocked. By adding relatively dense Al_2_O_3_ to a SiO_2_ substrate of low density a general systematic change of the surface area has to be considered as the specific surface area is normalized to mass (g). For clarification, adding 12.5% mass to a given quantity of SiO_2_ and assuming no change in the exposed surface area would counterintuitively result in a decrease of the mass related specific surface area from 506 m^2^ g^−1^ (see Table 1) to 450 m^2^ g^−1^ merely due to the added mass. The experimentally measured value here was 435 m^2^ g^−1^, in very good agreement with these considerations. In conclusion, the presented values for the surface area fairly represented the general trend, but the absolute values must be treated with reservation. The pore size distribution (see Figure 3) was calculated according to the BJH theory, which is based on the assumption of regular and cylindrical pores without any interconnection. Here again, the general trend of a decreasing pore size can be clearly seen in the calculated values. However, the absolute values were not reliable, as the silica substrate featured an irregular and unknown pore system. A reasonable assumption would be interconnected inkbottle-shaped pores formed by agglomeration of nano-sized primary particles.

**Figure 4:**
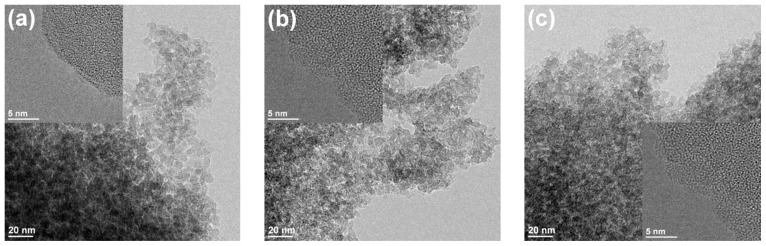
HRTEM images of (**a**) SiO_2_ reference, (**b**,**c**) after 1 and 3 ALD cycles with TMA and H_2_O at 200 °C.

The HRTEM images in Figure 4 show exemplary the uncoated and coated SiO_2_ particles after one and three cycles. The overall morphology and microstructure of the particles was retained during the ALD procedure at 200 °C and the AlO_x_ deposition. The primary particles of around 5 nm feature inner particle voids responsible for the high surface area of the material and the porosity. In general, electron microscopy of amorphous SiO_2_ in combination with Al is challenging, as they have close atomic numbers. Without advanced imaging techniques, (e.g., scanning transmission electron microscope) very low loadings of AlO_x_ in the surface cannot be observed. However, we did not detect full AlO_x_ layers or clusters. Consequently, the HRTEM images confirm a highly dispersed AlO_x_ on the surface. 

**Figure 5:**
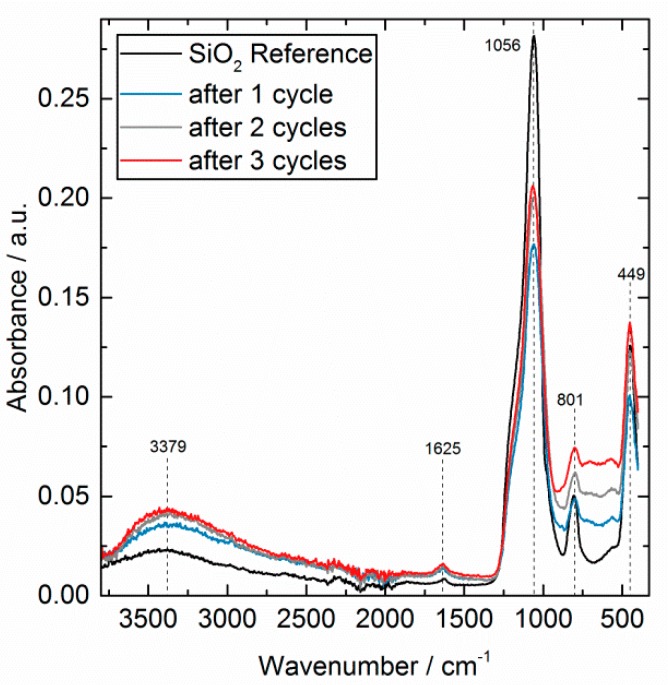
Absolute ATR-FTIR spectra of the SiO_2_ reference (black) and the AlO_x_ deposited samples after 1 cycle (blue), 2 cycles (grey), and three cycles (red) at 200 °C using TMA and H_2_O. Atmospheric background is subtracted.

The absolute ATR-FTIR spectra of the reference and the coated samples are presented in Figure 5 and prove the growth of an amorphous AlO_x_ on SiO_2_. All spectra showed three strong bands for SiO_2_ in the low frequency region (<1200 cm^−1^). The adsorption bands at 1056 cm^−1^ and 801 cm^−1^ are attributed to asymmetrical and symmetrical Si-O-Si stretching, and the band at 449 cm^−1^ to the corresponding Si-O bending vibrations [23]. By depositing AlO_x_ cycle by cycle, several broad features appeared in the range of 990–450 cm^−1^, without a notable change related to the position of the Si-O bands. Those new characteristic bands indicate bulk aluminum oxide and mixed Al/Si oxides. The bands superimpose with each other and the SiO_2_ bulk bands, so a specific band assignment is not appropriate. In general, Si-O-Al stretching vibrations appear usually between 1000–926 cm^−1^ and Al-O-Si bending vibrations in the range between 800–500 cm^−1^ [24].

The Al-O stretching and O-Al-O bending led to bands between 850–750 cm^−1^ and 750–650 cm^−1^, respectively. One small feature at 1625 cm^−1^ corresponds to the OH bending mode. This mode was not changed by the ALD deposition, which may indicate those OH sites are inaccessible and/or non-reactive for ALD. The OH stretching appeared very broad around 3379 cm^−1^ [25]. The new AlO_x_ deposit on the surface led to a quantitative and qualitative change in this mode as expected. Besides the Si-OH, the surface was populated with additional Al-OH groups during the ALD until a full AlO_x_ layer was formed after more cycles.

Possible residues of precursor material can stay on the substrate surface and lead to undesired contamination. We analyzed the carbon contamination in the samples by elemental analysis. Around 1.6% carbon uptake was found after one cycle at 200 °C. The amount of contamination stayed constant at 1.6% after two cycles and then decreases to 1.4% after three cycles. Most of the contamination was accordingly built-in with the first cycle of TMA. The exclusive carbon source in the process is the methyl group from the TMA. Incomplete reaction of the TMA ligand with H_2_O or trapped/attached carbonaceous species after the reaction with H_2_O can be safely assumed as the source of the measured residues. The powder surface structure may favor the development of contamination due to its complex porosity, in particular micropores [26]. Longer overall contact with H_2_O in the following cycles may help to clean the surface of TMA residues. 

### 3.2. Influence of Substrate Temperature

**Figure 6:**
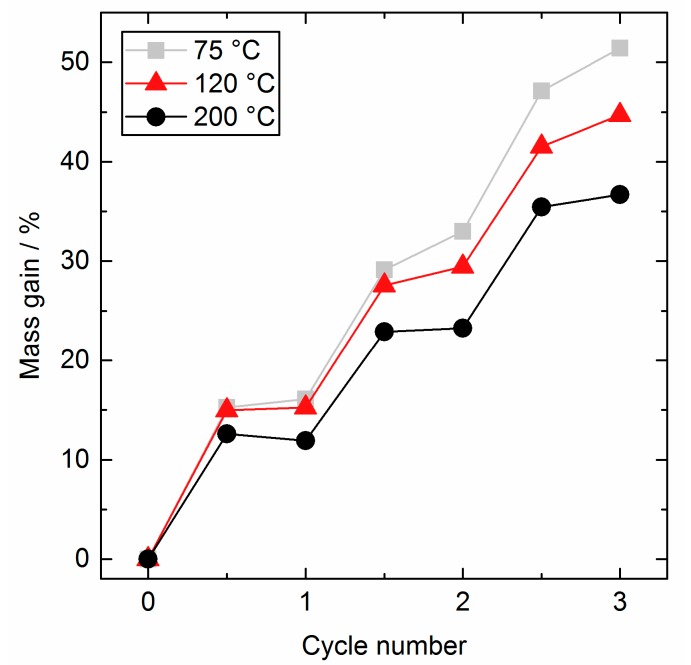
Mass gain over cycle number for AlO_x_ coating on SiO_2_ at different temperatures: 200 °C (black, ●), 120 °C (red, ▲), and 75 °C (grey, ■).

The AlO_x_ coating experiment was repeated at 75 °C and 120 °C to study the influence of the substrate temperature. Again, a self-terminating process was found for all temperatures through all three cycles. The percentage mass gain per half cycle is given in Figure 6. Full saturation curves are presented in the Supplementary Materials. 

**Figure 7:**
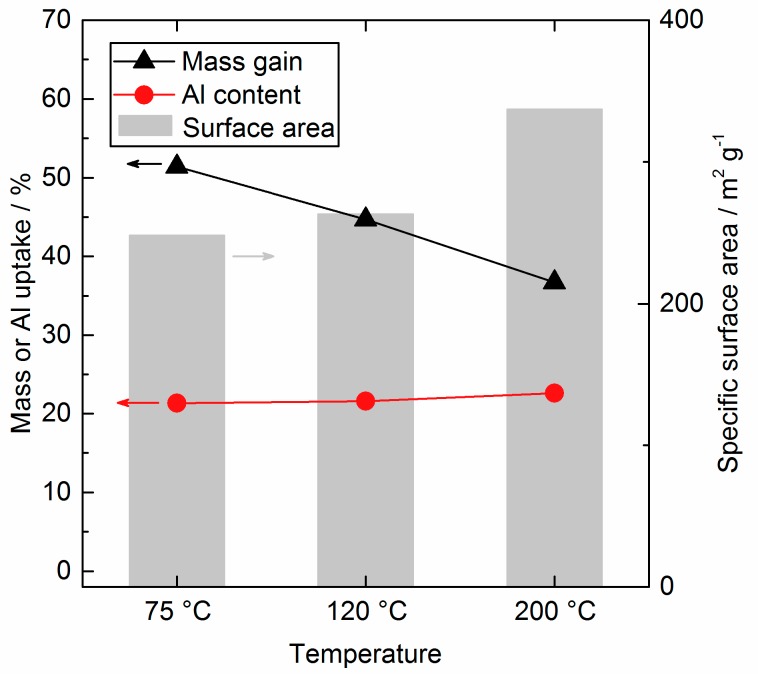
Specific surface area (grey, background, right axis), mass gain in wt % (black, left axis) and Al wt % (red, left axis) for different substrate temperatures after three cycles.

Lower substrate temperatures led to a higher mass gain per cycle and a sharper decrease of surface area, as displayed in Figure 7. The Al uptake increased from 21.3% for 75 °C to 21.6% for 120 °C and up to 22.6% for the 200 °C sample, but are comparable in a reasonable range. As it is discussed in literature, the total amount of deposited Al is significantly influenced by the amount of reactive surface sites [27]. As it was already mentioned, the SiO_2_ substrate was not profoundly changed in the working temperature range above 72 °C and we expect the same amount of SiO_2_ surface sites for all temperatures. However, the surface composition changes when AlO_x_ is deposited and different amounts and sorts of surface sites can evolve and their interaction will be influenced by temperature, e.g., more condensation reactions of new OH groups taking place at higher temperatures. The first cycle for all temperatures add up in a comparable mass gain as seen in Figure 6, as the precursor predominantly reacted with the selfsame SiO_2_ surface. The following cycles diverge much stronger as new surface sites were created. Lower mass gain indicates here an already more interconnected AlO_x_ for higher deposition temperatures and more isolated AlO_x_ species, containing more O and H atoms as well, on the surface of SiO_2_ at low temperatures, respectively. In other words, the AlO_x_ deposited at 200 °C was much denser than the AlO_x_ deposited at 75 °C. In general, literature reports about an inversely proportional relationship between the process temperature and the growth/mass gain for the TMA/H_2_O, owing to the temperature influence on the nature and amount of surface sites [13].

To compare our results with common other ALD studies, it is necessary to determine the average growth per cycle, which is generally easy for the deposition on flat substrates. When working with porous powders this value is not directly accessible. Here, the density of 3 g cm^−3^ at 200 °C from literature for the AlO_x_ deposit for all temperatures is applied to calculate the average layer thickness based on the overall mass gain. The resulting calculated growth rates are 0.81 Å/cycle for 200 °C, 0.98 Å/cycle for 120 °C, and 1.10 Å/cycle for 75 °C, and conform to other literature [28]. However, the assumption of a constant density is inaccurate.

**Figure 8:**
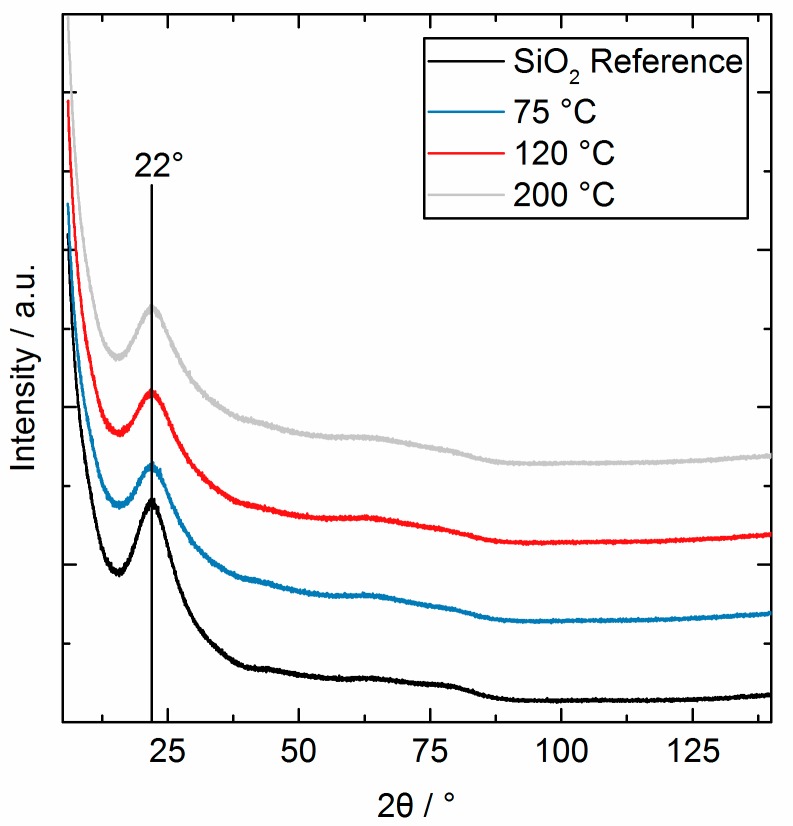
XRD pattern of the reference SiO_2_ and the AlO_x_ coated samples synthesized at different substrate temperatures after three cycles.

XRD shows (Figure 8), that the SiO_2_ powder and the as-deposited AlO_x_ coatings were amorphous for all temperatures. One broad reflex at 22° is attributed to amorphous SiO_2_. No crystalline ad phases or phase changes were found. 

When discussing three ALD cycles, the question of a closed monolayer of AlO_x_ on the surface arises. In general, this question is not easy to answer, due to the unknown structure and dimension of such a monolayer [29,30]. The very first layer of a new material on a well-defined crystal structure e.g., SiO_2_ crystal or Si wafer, most probably follows the already given crystallinity of the support material (epitaxy). The structure will morph from layer to layer and after a certain thickness finally result in the corresponding crystal structure of the deposit material depending on the process temperature and parameters, i.e., Al_2_O_3_. In this study, we coated amorphous SiO_2_ with an amorphous AlO_x_ and therefore cannot definitely rely on known structures. Thus, three different approaches for answering the question of the dimension of a monolayer are discussed below. 

Despite the fact that the investigated material is non crystalline, a reasonable starting point for a rough estimate of the space requirement of Al and O containing layer, can be a well-known Al_2_O_3_ structure, for instance the corundum structure, α-Al_2_O_3_ [31]. It has a hexagonal crystal lattice and is composed of AlO_6_ octahedra with O tetrahedrally coordinated by Al atoms. The unit cell repeats itself every six layers, as the basal plane features empty Al sites. In this unit cell, the thickness of one layer is 2.166 Å [32]. By combining the lattice parameters and the given fact of two Al atoms per layer (12 per unit cell), the concentration of Al per nm^2^ in such a layer can be calculated to theoretical 10.2 atoms_Al_ nm^−2^. Another estimate of the dimension of a monolayer is obtained from the density of the material. By assuming an isotropic and equilateral cube of AlO_1.5_ and a density of, again, 3 g cm^−3^ the space requirement of Al is 0.0926 nm^2^, giving 10.8 atoms_Al_ nm^−2^. This result is already comparable with the one deriving from the crystal lattice approach. To come back to the experimental data, the height of the deposited layer after three cycles was determined by the already calculated growth per cycle and laid between 2.4–3.3 Å depending on the substrate temperature. Here, the calculated thickness from the experimental data indicates the possibility of an already closed monolayer of AlO_x_. Still, it is important to keep in mind that the calculated thickness is inaccurate, because the exact density is unknown and most likely differs from the density of around 4 g cm^−3^ for α-Al_2_O_3_ [28] and the calculated height of 2.166 Å in a corundum. On the other hand, the Al surface concentration of our synthesized samples can be straightforwardly calculated from the Al content and the BET surface area. Though, the assumption of a two-dimensional growth of the AlO_x_ process needs to be made, meaning TMA preferably reacted with the original SiO_2_ surface rather than the AlO_x_ islands [33]. The Al-O species are consequently arranged side by side and not on top of each other. Thereby the experimental values for the surface concentration laid in the range of 8.9–9.9 atoms_Al_ nm^−2^ and would suggest insufficient Al atoms on the surface for a closed monolayer, irrespective of the above presented theoretical approach to dimension a monolayer. In conclusion, the question of a closed monolayer cannot certainly be answered. Three cycles are definitely on the edge to a closed layer, but might not be enough. Following the presented calculations, it can be safely assumed that less than three cycles will not give a monolayer of AlO_x_.

### 3.3. Scale Up in Fixed Bed

Samples FB.top, FB.middle, and FB.bottom were synthesized in the large fixed bed reactor using three AlO_x_ ALD cycles with TMA and H_2_O. The in situ mass gain was consequently not recorded. Yet, QMS measurement confirmed the changing gas atmosphere and breakthrough curves for all cycles, as it is presented elsewhere [15]. The fixed bed consisted of around 10 g SiO_2_ powder (10× more, than in the balance) and was segmented in three partitions to investigate the homogeneity of the coating through the entire bed length. The surface area and the wt % Al for all segments corresponds to the 200.3c sample (see Table 1), that was synthesized in the balance with identical process parameters. Therefore, we can conclude homogeneity of all samples along the bed, as well as a homogeneously deposition of AlO_x_. In addition, the samples showed nearly no carbon contamination, in contrast to the balance samples. Presumably, the gas-solid contact in the large fixed bed reactor was improved compared to the balance system, leading to a lower carbon contamination. This is rationalized by the frequent interruption of the balance system, due to the lifting of the crucible for the mass measurement. During this measurement, the gas-solid contact was largely minimized and most of the precursor bypasses the sample material.

## 4. Conclusions

We reported about the growth of AlO_x_ via 1–3 ALD cycles of TMA/H_2_O on mesoporous SiO_2_ particles. By applying an average density for all deposits, the growth per cycle was calculated to 0.81 Å/cycle for 200 °C, 0.98 Å/cycle for 120 °C, and 1.10 Å/cycle for 75 °C, and is in good agreement with other AlO_x_ processes reported. The in situ gravimetry is a convenient tool to observe the saturation behavior on the substrate surface during the ALD process itself. We verified a self-limiting deposition for all temperatures, half-cycles, and cycles. As the first ALD cycles proceed on the SiO_2_ surface and not on an Al_2_O_3_, the overall mass gain and its distribution between the TMA and H_2_O cycle changed from cycle to cycle. We conclude predominantly a two-ligand exchange reaction of the TMA molecule with the surface OH groups for the first cycle. With increasing cycle number, more single-ligand exchange was taking place. The Al content is insignificantly influenced by the substrate temperature. The higher mass gain for lower temperatures was consequently based on higher incorporation of O and H in the deposit, leading to a less dense AlO_x_ layer. The very unique feature of ALD to coat high surfaces including highly porous substrates in a conformal manner was proven by nitrogen adsorption experiments. Different approaches to estimate a possible amorphous monolayer of AlO_x_ on the SiO_2_ surface were discussed and we can safely assume that three ALD cycles were not enough to form this monolayer. Finally, we demonstrated the compelling advantage of the in situ gravimetric monitoring for ALD on powder materials. Further analysis of the TMA/H_2_O and other ALD processes on other challenging powder substrates, e.g., catalysts, are of high interest and will be the subject in future investigations. 

## Figures and Tables

**Table 1:** Sample family of SiO_2_ powder coated samples with AlO_x_ using TMA and H_2_O.

Description	Number of Cycles	Reactor Temperature/°C	Mass Uptake ^+^ − Cycle 1/%	Mass Uptake ^+^ − Cycle 2/%	Mass Uptake ^+^ − Cycle 3/%	Mass Uptake ^+^ − Total/%	Specific Surface Area/m^2^ g^−1^	Al Content/wt %	Al Uptake ^+^/wt %	C Content/wt %	C Uptake ^+^/wt %
SiO_2_ reference	-	-	-	-	-	-	506	0.0	0.0	0.00	0.00
200.1c	1	200	12.5	-	-	12.5	435	8.0	8.7	1.52	1.55
200.2c	2	200	13.4	11.6	-	24.0	383	13.7	15.9	1.59	1.62
200.3c	3	200	11.9	11.4	13.4	36.6	337	18.4	22.6	1.33	1.35
120.3c	3	120	15.2	14.2	15.2	44.6	263	17.8	21.6	2.43	2.50
75.3c	3	75	16.1	16.9	18.4	51.4	248	17.6	21.3	2.66	2.74

^+^ Mass uptake, Al and uptakes are given as the percent uptake compared to the original substrate.

**Table 2:** Scale-up samples of SiO_2_ powder coated samples after 3 cycles at 200 °C using TMA and H_2_O.

Description	Specific Surface Area m^2^ g^−1^	Al Content wt %	C Content wt %
200.3c	337	18.4	1.33
FB.top	311	16.8	0.02
FB.middle	311	17.2	0.00
FB.bottom	305	15.8	0.00

## Supplementary Materials

The following are available online at http://www.mdpi.com/2079-4991/8/6/365/s1, Figures S1, S2, and S3: In situ gravimetric monitoring of three cycles TMA/H_2_O on SiO_2_ particles at 200 °C/120 °C/75 °C.
