# Supplementary material for: Investigating the Trimethylaluminium/Water ALD Process on Mesoporous Silica by In Situ Gravimetric Monitoring
Source: Nanomaterials (Basel). 2018 May 24;8(6):365. doi: 10.3390/nano8060365 (PMC6027410; doi:10.3390/nano8060365)
Supplement: Supplementary file 1 [file nanomaterials-08-00365-s001.pdf]

nanomaterials

Supplemental

Investigating the trimethylaluminium/water ALD process on mesoporous silica by *in situ* gravimetric monitoring

V. E. Strempel[1], K. Knemeyer[1], R. Naumann d'Alnoncourt[1,*], M. Driess[1,2]and F. Rosowski[1,3]

[1] BasCat - UniCat BASF JointLab, Technische Universität Berlin, Hardenberstraße 36, 10623, Berlin, Germany
[2] Institut für Chemie. Technische Universität Berlin, Straße des 17. Juni 135, 10623, Berlin, Germany
[3] Process Research and Chemical Engineering, BASF SE, Carl-Bosch-Straße 38, 67056 Ludwigshafen, Germany

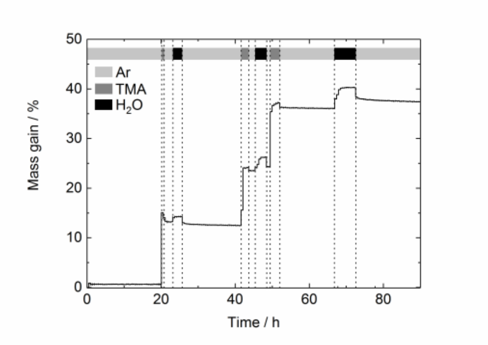

Figure S1. In situ gravimetric monitoring of three cycles TMA/H_2O on SiO_2 particles at 200°C. Dosing procedure pictured in the upper part.

Deleted: 3

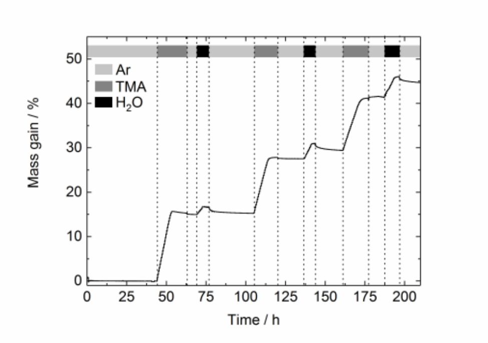

Figure S2. In situ gravimetric monitoring of three cycles TMA/H_2O on SiO_2 particles at 120°C. Dosing procedure pictured in the upper part.

Deleted: 3

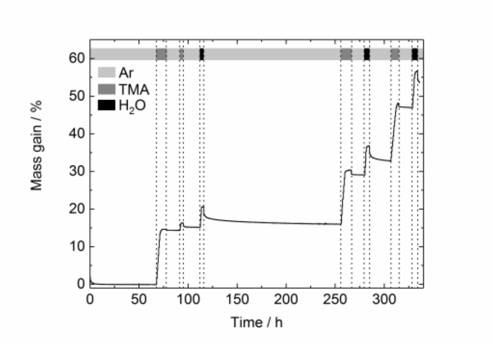

Figure S3. In situ gravimetric monitoring of three cycles TMA/H_2O on SiO_2 particles at 75°C. Dosing procedure pictured in the upper part.

Deleted: 3

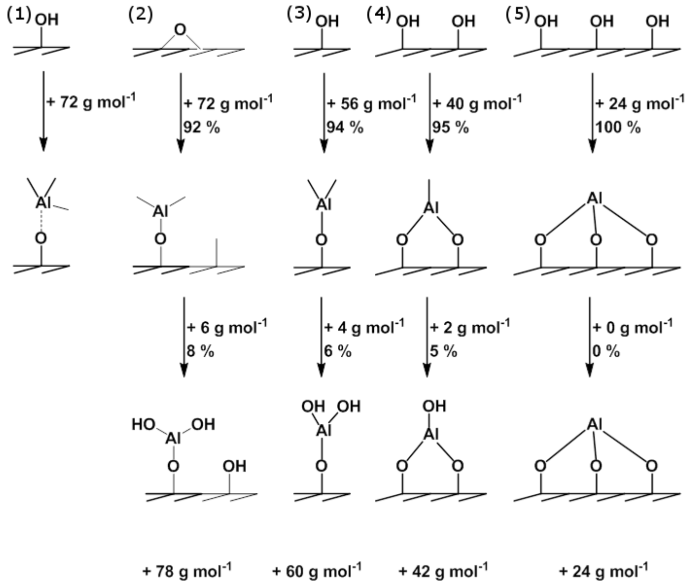

Figure S4. Possible surface reactions during TMA and H_2O half-cycles and the corresponding mass gains per mol (reaction from top to bottom): (1) Associative reaction, (2) dissociative reaction, (3) single ligand exchange, (4) double ligand exchange, and (5) triple ligand exchange.

Deleted: a
